# Growth charts of Brazilian girls with Turner syndrome without the use of GH or oxandrolone

**DOI:** 10.1016/j.jped.2024.09.003

**Published:** 2024-10-25

**Authors:** Stela Carpini-Dantas, Gil Guerra-Junior, Andréa Trevas Maciel-Guerra, Denise Barbieri Marmo, Tarsis Paiva Vieira, Carolina Paniago Lopes, Maria Tereza Matias Baptista, André Moreno Morcillo, Sofia Helena Valente de Lemos-Marini

**Affiliations:** a Departamento de Pediatria da Divisão de Endocrinologia Pediátrica, Universidade Estadual de Campinas (UNICAMP), Faculdade de Ciências Médicas (FCM), Campinas, São Paulo, Brazil; b Departamento de Medicina Translacional, Divisão de Genética Médica e Medicina Genômica, Universidade Estadual de Campinas (UNICAMP), Faculdade de Ciências Médicas (FCM), Campinas, São Paulo, Brazil; c Departamento de Medicina Clínica, Divisão de Endocrinologia, Universidade Estadual de Campinas (UNICAMP), Faculdade de Ciências Médicas (FCM), Campinas, São Paulo, Brazil

**Keywords:** Growth chart, Turner syndrome, Body height, Body weight, Disorders of sex development

## Abstract

**Objective:**

The development of specific growth charts for Turner Syndrome (TS) promotes adequate assessment of growth and weight gain, and earlier diagnosis of comorbidities, and may help to analyze the effectiveness of treatments to promote growth and puberty. The aim of this study was to construct a growth chart with the largest possible series of patients with a cytogenetic diagnosis of TS from a Brazilian reference center.

**Methods:**

This is a longitudinal study, with 259 cases of TS born between 1957 and 2014 and followed between 1975 and 2019, without the use of GH or oxandrolone. 3,160 height measurements and 2,918 wt measurements were used, with subsequent calculation of the Body Mass Index (BMI). For data analysis, the “GAMLSS” package of the “R” software was used.

**Results:**

The mean target height was 157.8 cm (standard deviation 5.2; median 160.4 cm). The mean height of patients with TS at 20 years of age was 145.6 cm (standard deviation 5.9; median 146.7 cm). Height, weight, and BMI by age graphs were developed for TS girls between 2 and 20 years.

**Conclusion:**

These growth charts may be used to monitor the growth of girls with TS and to verify the effect of adjuvant treatments on promoting growth.

## Introduction

Assessing a child's weight and height gain over time is essential for monitoring their health status. Furthermore, both are directly influenced by genetic load and environmental factors.^1^

Turner Syndrome (TS) is cytogenetically characterized by the presence of an X chromosome and partial or total loss of the second sex chromosome.^2^ Short stature, the most prevalent sign, is present in approximately 95 % of cases.^3^ The adult height of these women is, on average, 20 cm shorter than expected for the respective population.^4^, ^5^, ^6^, ^7^ The mean adult height of patients with TS without GH treatment was 142.1 cm in France,^8^ 146.8 cm in Germany,^6^ 141.3 cm in Japan,^9^ 137.9 cm in Argentina^10^ and 144.8 cm in Brazil,^5^ while mean adult height of the general female population was 160.9,^8^ 164.6,^6^ 158.2,^9^ 160.7^10^ and 162.5 cm,^5^ respectively.

Short stature in TS is explained, among other factors, by haploinsufficiency of the SHOX gene.^11^^,^^12^ Furthermore, since fetal life there is an acceleration in the process of gonadal degeneration^13^ and, although 30 to 50 % of patients still present some degree of spontaneous pubertal development,^14^^,^^15^ they are poorly exposed to estrogens, which negatively influences the axis GH-IGF-1, linear and longitudinal growth, growth spurt and, consequently, adult height.^16^^,^^17^ There is also a higher incidence of autoimmune diseases, such as thyroiditis and celiac disease, which can also compromise height.^12^

In TS, growth differs from the normal population regarding growth patterns throughout childhood and adult height.^7^ The variation in height in relation to the standard, in addition to facilitating the early diagnosis of comorbidities, may be a good tool for analyzing the efficiency of growth promotion treatments, justifying the development of a specific growth chart for this clinical condition.^9^^,^^18^ Growth charts for patients with TS are available in the literature, but they present great heterogeneity in terms of statistical and data collection methods, total sample number and ethnic origin of the studied population.^6^, ^7^, ^8^, ^9^^,^^19^, ^20^, ^21^

Reviews published by Bertapelli et al.^21^ in 2014 and by Isojima et al.^7^ in 2023 evaluated the specific growth charts for TS available in the literature and compared the methodological and statistical aspects of each of them, revealing heterogeneity among existing publications. In Brazil's reference services, TS growth charts published by Ranke et al. in 1983^6^ or by Lyon et al. in 1985^19^ have been used, since there is not a Brazilian chart for this condition published in the literature.^21^

The aim of this study was to develop specific growth charts for TS based on serial measurements of weight and height of a group of Brazilian patients followed at a reference medical service without the use of GH and/or oxandrolone.

## Methods

This is a retrospective longitudinal study of TS patients with confirmed cytogenetic diagnosis.

The authors included all patients with TS born between 1957 and 2014 and followed in the Pediatric Endocrinology Outpatient Clinic of the Clinical Hospital from the State University of Campinas (UNICAMP), Campinas, Brazil between 1975 and 2019. The clinical follow-up is systematized in the service. Part of the team, including the physician responsible for the service, monitored all patients throughout the evaluation period. The medical appointments were usually scheduled every 6 mo. The presence of autoimmune diseases (e.g., thyroiditis and celiac disease) and cardiopathy were routinely investigated and recommendations were given to prevent obesity and promote health (nutrition, hygiene, and physical activity).

All medical records of each patient's height and weight measurements were obtained from their first appointment at the service until 2019. The measurements had been taken by the nursing team or medical staff at the pediatric clinic, often by both.^22^ The Body Mass Index (BMI) was calculated from simultaneous measurements of weight and height (BMI = weight [Kg]/height^2^ [m^2^]). The entire team that carried out the measurements over these years had prior training.

Complementary data such as birth weight and length, parents' height, presence of comorbidities, and medications used were also obtained. Most of the parents' heights were determined in the pediatric clinic or in health units. The target height was calculated from the parents' height ([mother's height + father's height – 13] ÷ 2) (in cm).^1^

Patients were excluded from the study if they were over 20 years old at the first appointment, <2 years old at the last appointment, or if there were no anthropometric measurements. Those with conditions with direct implications on growth other than TS itself, such as severe chronic diseases, and those with previous use of GH or oxandrolone were also excluded. Of the patients who underwent rhGH/Oxandrolone for growth promotion during follow-up, measurements obtained after the start of GH or oxandrolone were excluded.

The use of hormone replacement therapy with estrogens and progestins and low birth weight were not discriminated in this study.

For statistical analysis, the SPSS^23^ software and the Generalized Additive Models for Location Scale and Shape (GAMLSS)^24^ package from the “R” software^25^ were used. Iterative procedures like those carried out by the World Health Organization (WHO) were adopted in the development of the growth charts.^26^ The identification and removal of atypical data was carried out by visual inspection of the graphs.

The Box-Cox Power Exponential and Box-Cox Cole and Green distributions were initially selected to develop the charts. To evaluate the models, Akaike Information Criterion (AIK) and Generalised Akaike Information Criterion (GAIC) were used.^27^

After determining the best transformation “λ” for age (*x* = ageλ), the most appropriate degrees of freedom (df) for the parameters (µ, σ, ν, τ) of the distributions were iteratively determined. Finally, the residuals were analyzed using the worm-plot graph and the Q test, that is until the lowest value of AIC and GAIC was obtained and resulted in normally distributed residuals with zero mean. The charts were smoothed using cubic splines.^26^ The Box-Cox Power Exponential distribution proved to be more suitable for developing the three chart models.

Once the most appropriate distribution and model for constructing the chart had been defined, the smoothed percentiles 3, 10, 25, 50, 75, 90, and 97 were determined and graphs were constructed.^26^ This format was chosen because growth curves are frequently used in both medical care and research.

This study is in accordance with the Declaration of Helsinki and the protocol was approved by the Research Ethics Committee (931/2008). The study was exempt from informed consent as it was retrospective and non-interventional.

Data from this study can be requested from the authors at any time**.**

## Results

The case series initially included 310 patients, of which 51 (15 %) were excluded for not meeting the inclusion criteria: 17 for being over 20 years old at the first appointment, 2 for being under 2 years old at the last consultation, 2 for lack of anthropometric measurements, 15 due to severe chronic illness and 15 due to previous use of GH or oxandrolone.

The growth charts were constructed based on the measurements of 259 patients, born between 1957 and 2014 (median 1989), whose follow-up took place between 1975 and 2019. The age at which the assessment began varied from 2 to 20 years (mean 9.3 years, standard deviation 5.7 years, median 14.4 years).

The karyotype was 45, X in 120 cases. Of the 58 cases with chromosomal mosaicism without structural aberrations, the most frequent was 45, X/46,XX (*n* = 46), and among the 68 karyotypes with structural aberrations, with or without mosaicism, the most frequent was 45, X/46,X, i(Xq) (*n* = 22). The presence of Y chromosome material was detected in 26 patients, nine of them identified in the karyotype and 17 with subsequent genetic tests, such as polymerase chain reaction or fluorescent in situ hybridization (Supplement 1).

At birth, the mean weight (*n* = 218) was 2,800 g and the mean length (*n* = 176) was 46.1 cm. In this sample, the mean height of mothers (*n* = 222) was 158.0 cm and that of fathers (*n* = 209) was 170.4 cm. The calculated mean target height (*n* = 208) was 157.8 (Table 1).

**Table 1:** Clinical characteristics of the 259 patients evaluated in the study.

Variable	n	Mean ± SD	Variation	Median
Birth weight (kg)	218	2.8 ± 0.5	1.1–4.2	2.8
Length at birth (cm)	176	46.1 ± 3.0	31–53	47.0
BMI at birth (kg m²)	176	13.1 ± 1.7	8.6–19.8	12.9
Final height^a^ (cm)	38	145.6 ± 5.9	130.9–156.8	146.7
Mother's height (cm)	222	158.0 ± 6.8	141.8–173.9	158.0
Father's height (cm)	209	170.4 ± 6.8	150.0–196.0	170.0
Target Height^b^	208	157.8 ± 5.2	144.9–176.5	160.4

n, number of measurements available.

a Final Height is considered the last height measurement observed in patients aged 20 or over.

b Target Height Calculation based on the concept of Tanner et al.^1^

Information about puberty was available for 202 patients, whose ages were over 13 years during the evaluation. Of the 59 (29 %) girls with some degree of spontaneous puberty, 27 of them had not used hormone replacement therapy (HRT) with estrogens and/or progestins until the last record collected because they did not need it until the last evaluation or because they were still too young to start treatment.

In total, 3160 height measurements and 2918 wt measurements were selected, from which the BMI was calculated. The number of height observations per patient ranged from 1 to 38, with most patients (*n* = 187, 72 %) having >6 observations; in almost half of the sample (*n* = 121, 46.7 %) there were >11 measurements per patient included to create the chart. Only 14 patients (5.4 %) contributed with a single height measurement and 15 patients (6.1 %) with a single weight measurement (Supplement 2)

The number of measurements also varied depending on the age. The highest number of height measurements (*n* = 1695 - 53.7 %) was obtained between 13 and 20 years of age. (Supplement 3).

At the last appointment, only 38 (14.6 %) girls had already reached 20 years of age. The mean height of these girls was 145.6 cm ± 5.9 cm and the median was 146.7 cm. After age 20, in 6 girls whose karyotype contained Y chromosome material, the mean adult height was 147.2 cm ± 6.1 cm and the median was 147.4 cm. In 15 girls with complete spontaneous puberty (spontaneous menarche), the mean adult height was 147.6 cm ± 5.8 cm and the median was 148.6 cm. The mean and median heights after age 20, according to the year of birth, were respectively 144.9 cm ± 6.0 cm and 145.5 cm (*n* = 21) between 1957 and 1989, and 146.4 cm ± 5.9 cm and 147.0 cm (*n* = 17) between 1990 and 1999.

The actual distributions of height, weight and BMI by age were plotted in the graphs shown in Supplement 4.

**Figure 1:**
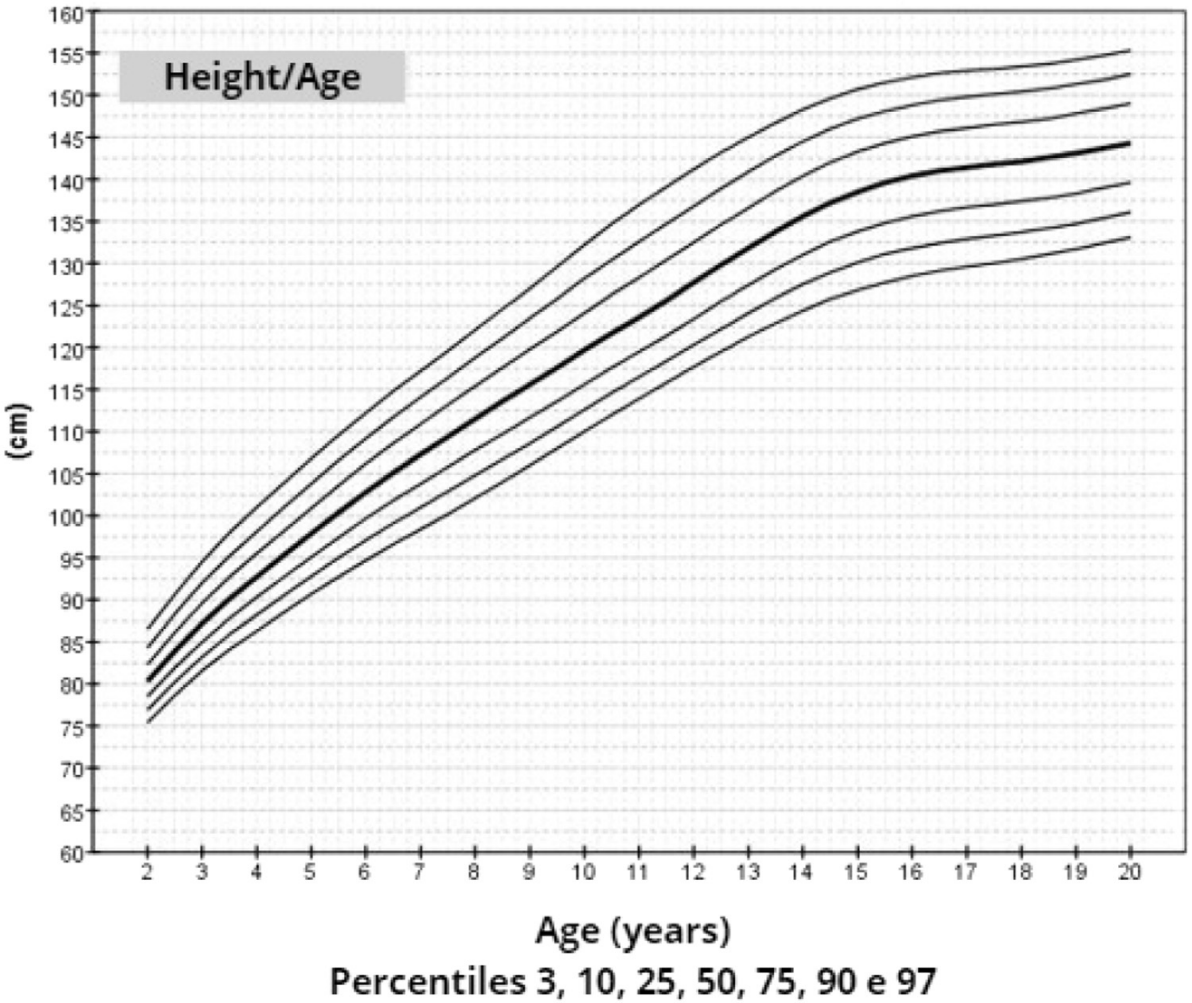
Height-for-age chart of 259 patients with TS.

**Figure 2:**
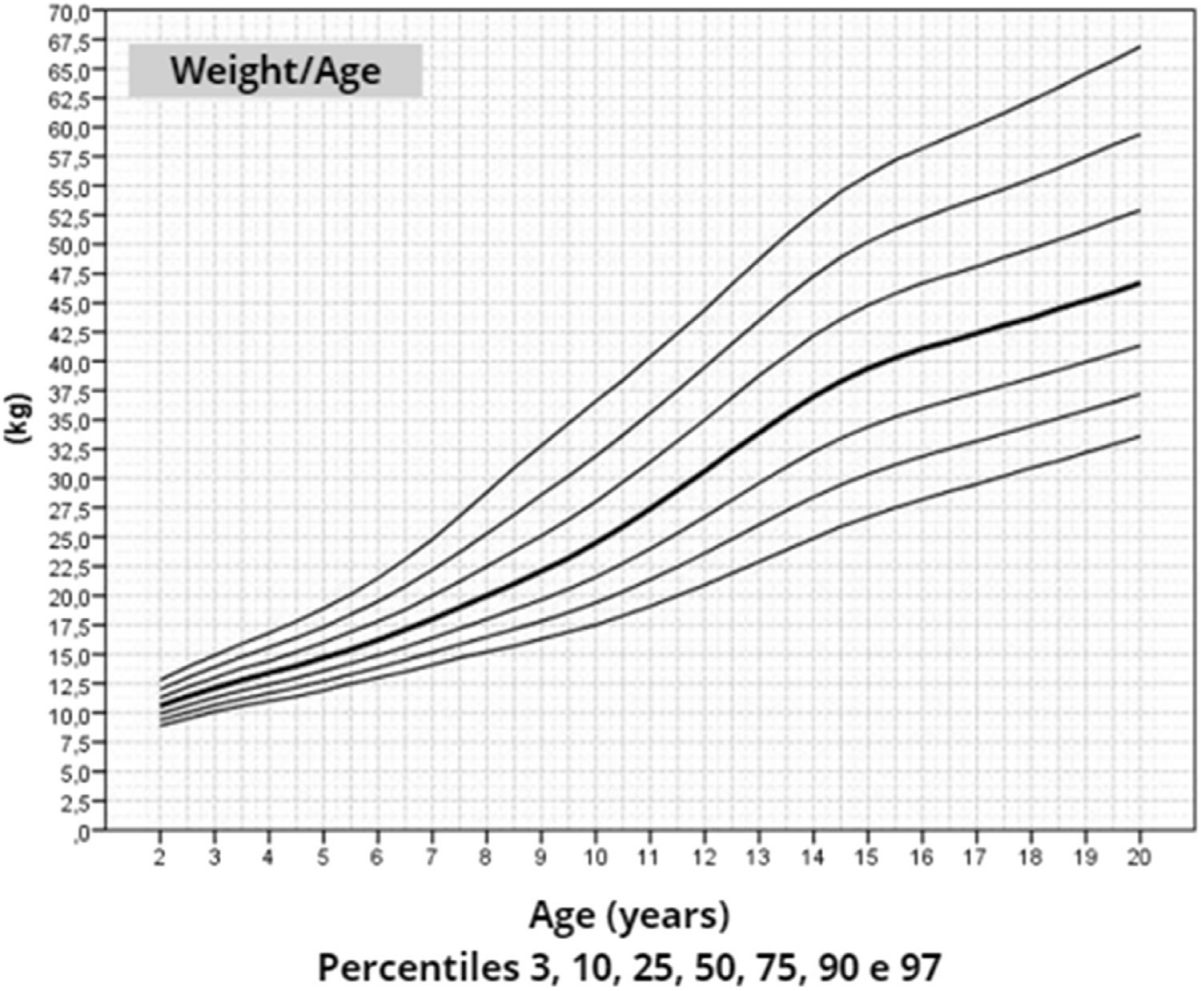
wt-for-age chart of 259 patients with TS.

**Figure 3:**
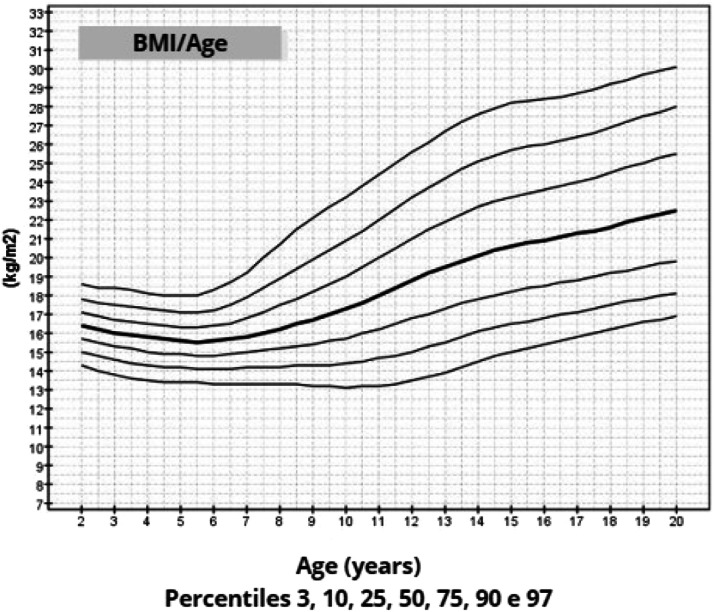
BMI-for-age chart of 259 patients with TS.

After data analysis, charts of height (Figure 1), weight (Figure 2) and BMI (Figure 3) by age were obtained. The weight, height and BMI values found by percentile and standard deviation are found in Supplements 5, 6 and 7, respectively.

## Discussion

To improve the care of children with TS, the spontaneous growth of patients treated in a systematic and homogeneous manner was studied, and growth charts of Brazilian girls with TS were constructed based on the statistical method recommended by the WHO and with data reliability analysis.

The data used to construct the growth charts were obtained from 259 patients who had a diagnosis confirmed by karyotype. The most frequent were 45,X (46 %) and 45,X/46,XX (18 %). The group used to construct the chart published by Sempé et al. in 1996 had a similar distribution^8^: 48 % were 45,X and 26 % 45,X/46,XX. A Previous study published by Ranke et al.^6^ found 60 % with a 45,X karyotype and 15 % with 45,X/46,XX. A hypothesis for the progressively smaller number of 45,X karyotypes over the years is the evaluation of a higher number of metaphases over time and the improvement in the technical quality of karyotype.^28^

This series consisted of 259 patients, followed up in the same service, a higher number than obtained Ranke et al.^6^ = (6) and Sempé et al.^8^ who evaluated 150 (Germany) and 167 (France) cases, respectively. Available charts with larger samples include one proposed by Lyon et al.,^19^ comprising 459 patients from various countries (Germany, France and Finland) or have an ethnic origin very different from that of Brazil, such as that constructed by Isojima et al.^9^ with information from 1565 Japanese girls.

For the construction of growth charts, not only the total number of patients is important, but also the number of measurements obtained. About 3160 height measurements were used in this study (259 girls), while Ranke et al.,^6^ Lyon et al.^19^ and Isojima et al.^9^ used 384 (150 girls), 2.214 (459 girls) e 5.772 (1.565 girls) measurements, respectively.

In this study, the initial proposal was to assess the growth of girls born in the last 30 years. However, in order to use the largest possible number of patients and measurements, and since TS has a limited incidence in the population, it was decided to include all patients born between 1957 and 2014 and followed in the service from 1975 to 2019. There is not much data in the literature on the secular growth trend in the Brazilian population, and the total number of girls with height data above 20 years of age (*n* = 38) is too small for comparisons to define an increase in the secular growth trend in TS. Even so, the difference between the mean height after 20 years of age of patients born between 1957 and 1989 and between 1990 and 1999 was only 1.5 cm.

The mean adult height of the six girls with Y material was higher than that found in the group as a whole (147.2 cm × 145.6 cm), however, the low number of girls who had adult height does not allow us to carry out any statistical analysis, as has been demonstrated in other studies.^6^ The same occurred with the 15 girls who had complete spontaneous puberty (147.6 cm × 145.6 cm). It should be noted that the number of cases in these two groups was small, which makes it difficult to draw a final conclusion.

The adult height average in this study, 145.6 cm, was similar to Rongen-Westerlaken et al. 146.9 cm,^29^ Naera et al. 146.8 cm,^30^ Ranke et al.^6^ 146.8 cm, and higher than Lyon et al.^19^ 142.9 cm, Sempe et al.^8^ 142.1 cm, Isojima et al.^20^ 141.2 cm and Garcia-Rudaz et al.^10^ 139.8. Although the adult height (145.6 cm) is 4 cm higher than Isojima et al.,^20^ the calculated average weight was equivalent, 46.7 kg and 46.6 kg,^20^ respectively, and lower than that presented by Ranke et al.^6^ (52.3 kg). Furthermore, the mean BMI was 22.5 and varied from 16.9 to 30.1, demonstrating great variability in the body composition of the girls in this sample. Furthermore, the analysis of the height-for-age chart shows that there seems to be more elongated growth after the age of 18, which is probably due to the late induction of puberty, as also suggested in other studies.^7^

As it was a study carried out in a single reference service for TS, with part of the team present at all appointments, there is greater homogeneity of data and monitoring of the girls evaluated, including rigor in obtaining anthropometric measurements. Therefore, despite the total number of patients being relatively lower than in multicenter studies, there is greater reliability in the measurements used to create the growth charts. The studies published by Ranke et al.^6^, Lyon et al.^19^, Sempé et al.^8^ and Isojima et al.^9^ evaluated measurements obtained from at least 2 different centers, including information obtained from databases fed by professionals working in several different regions.^9^^,^^20^

In this study, the largest number of height measurements per patient was obtained between 13 and 20 years of age, unlike other published studies,^8^^,^^20^^,^^29^^,^^30^ in which it was difficult to obtain real height after 10 years of age, including the use of mathematical formulas to calculate probable height in this age group.^8^ In total, 101 girls over 19 years of age were evaluated, twice the number evaluated by Rongen-Westerlaken et al.^29^ The recommendation for the use of GH in TS^12^ limits the number of measurements available without promoting growth,^7^^,^^12^ therefore, the future development of growth charts based on heights without pharmacological intervention in TS is unlikely.

According to the WHO, the ideal method for constructing growth charts is the longitudinal study.^9^^,^^26^ In this regard, only 5 % of the studied patients contributed with a single height measurement. In addition, most patients (72 %) contributed with >6 observations, reaching up to 38 height measurements of a single girl throughout her entire follow-up. There are studies available in the literature with a mixed longitudinal design,^6^^,^^9^ and others, such as the one published by Lyon et al.^19^ and Naeraa & Nielsen,^30^ which do not present the data collection method for comparison.

In this way, height, weight and BMI-for-age charts were constructed for a significant number of girls, with a cytogenetic diagnosis of TS, reliable anthropometric measurements, mainly longitudinal evaluation, using a statistical method suitable for smoothing and in accordance with WHO recommendations for constructing growth charts.^9^^,^^26^

The growth charts constructed in this study may be used to monitor growth, identify comorbidities earlier and evaluate treatments to promote growth in patients with TS.

## Conflicts of interest

The authors declare no conflicts of interest.

## Appendix Supplementary materials

Image, application 1
