# Supplementary material for: Growth charts of Brazilian girls with Turner syndrome without the use of GH or oxandrolone
Source: J Pediatr (Rio J). 2024 Oct 25;101(2):231–7. doi: 10.1016/j.jped.2024.09.003 (PMC11889696; doi:10.1016/j.jped.2024.09.003)
Supplement: Supplementary file 1 [file mmc1.docx]

**JPED-D-24-00287_Supplementary Materials**

**Supplement 1**

**Distribution of karyotypes of the 259 patients included in the study.**

| **Karyotype** | **Number of cases** | **Percentage of total (%)** |
| --- | --- | --- |
| 45,X | 120 | 46.3 |
| Mosaicism without structural aberration | 58 | 22.4 |
| 45,X/46,XX | 46 | 18 |
| 45,X/46,XY | 7 | 2.5 |
| 45,X/47,XYY | 3 | 1 |
| 45,X/47,XXX | 2 | 1 |
| Structural aberrations with or without mosaicism | 68 | 26.3 |
| 45,X/46,X,i(Xq) | 22 | 8 |
| 46,X,i(Xq) | 13 | 5 |
| 45,X/46,X,+mar (Y-) | 12 | 5 |
| 45,X/46,X,r(X) | 8 | 3 |
| 45,X/46,X,+mar (Y+) | 4 | 1.5 |
| 45,X/46,X,del(X) | 4 | 1.5 |
| 45,X/46,X,r(X)/46,XX | 2 | 1 |
| 45,X/46,X,i(Xq)/46,XX | 2 | 1 |
| 45,X/46,X,+mar | 1 | 1 |
| Others | 13 | 5 |
| Total | 259 | 100 |

**Supplement 2**

**Number of patients per number of observations of 259 girls with TS.**

| Number of measurements | Height | | Weight | | BMI | |
| --- | --- | --- | --- | --- | --- | --- |
|  | n | % | n | % | n | % |
| 1 | 14 | 5.4 | 15 | 6.1 | 15 | 6.1 |
| 2 | 13 | 5.0 | 12 | 4.9 | 12 | 4.9 |
| 3 | 15 | 5.8 | 14 | 5.7 | 14 | 5.7 |
| 4 | 16 | 6.2 | 15 | 6.1 | 15 | 6.1 |
| 5 | 14 | 5.4 | 13 | 5.3 | 13 | 5.3 |
| 6 a 10 | 66 | 25.5 | 65 | 26.5 | 65 | 26.5 |
| 11 a 15 | 33 | 12.7 | 32 | 13.1 | 32 | 13.1 |
| 16 a 20 | 33 | 12.7 | 29 | 11.8 | 29 | 11.8 |
| 21 a 25 | 27 | 10.4 | 25 | 10.2 | 25 | 10.2 |
| 26 a 30 | 22 | 8.5 | 20 | 8.2 | 20 | 8.2 |
| 31 a 38 | 6 | 2.3 | 5 | 2.0 | 5 | 2.0 |

**Supplement 3**

**Number of height and weight measurements by age group of 259 girls with TS.**

| **Age (Years)** | **Height** | **Weight** |
| --- | --- | --- |
| 2 \|--- 3 | 75 | 70 |
| 3 \|--- 4 | 75 | 63 |
| 4 \|--- 5 | 88 | 81 |
| 5 \|--- 6 | 99 | 89 |
| 6 \|--- 7 | 87 | 74 |
| 7 \|--- 8 | 100 | 96 |
| 8 \|--- 9 | 131 | 119 |
| 9 \|--- 10 | 153 | 144 |
| 10 \|--- 11 | 196 | 179 |
| 11 \|--- 12 | 229 | 210 |
| 12 \|--- 13 | 232 | 214 |
| 13 \|--- 14 | 248 | 230 |
| 14 \|--- 15 | 253 | 234 |
| 15 \|--- 16 | 263 | 241 |
| 16 \|--- 17 | 260 | 236 |
| 17 \|--- 18 | 242 | 227 |
| 18 \|--- 19 | 223 | 211 |
| 19 \|---\| 20 | 206 | 200 |
| Total | 3160 | 2918 |

**Supplement 4**


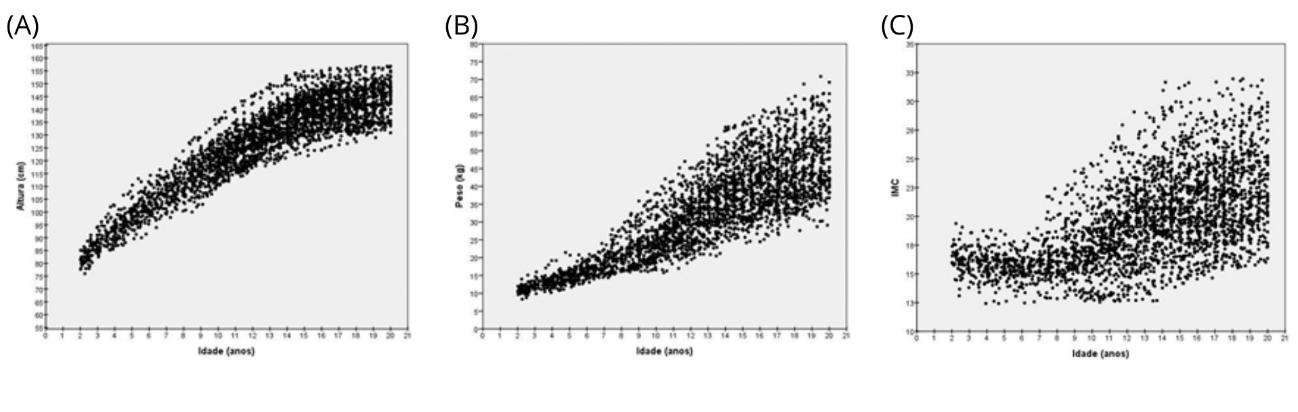
**Real distribution graphs of height, weight and BMI by age of 259 girls with TS.**

**Subtitle:** (A) Distribution of height by age. (B) Weight distribution by age. (c) Distribution of BMI by age.

**Supplement 5**

**Height calculated according to age group and percentile of 259 girls with TS.**

| Height (cm) by Age (years) | | | | | | | |
| --- | --- | --- | --- | --- | --- | --- | --- |
| Age | P3 | P10 | P25 | P50 | P75 | P90 | P97 |
| 2.0 | 75.4 | 76.9 | 78.5 | 80.3 | 82.3 | 84.3 | 86.5 |
| 2.5 | 78.6 | 80.2 | 81.9 | 83.9 | 86.1 | 88.3 | 90.6 |
| 3.0 | 81.6 | 83.2 | 85.0 | 87.2 | 89.6 | 92.0 | 94.5 |
| 3.5 | 84.1 | 85.9 | 87.8 | 90.1 | 92.7 | 95.2 | 98.0 |
| 4.0 | 86.3 | 88.2 | 90.3 | 92.7 | 95.5 | 98.1 | 101.0 |
| 4.5 | 88.5 | 90.5 | 92.7 | 95.3 | 98.2 | 101.0 | 103.9 |
| 5.0 | 90.7 | 92.8 | 95.1 | 97.9 | 100.9 | 103.8 | 106.8 |
| 5.5 | 92.7 | 95.0 | 97.4 | 100.4 | 103.6 | 106.6 | 109.6 |
| 6.0 | 94.7 | 97.1 | 99.7 | 102.8 | 106.2 | 109.2 | 112.2 |
| 6.5 | 96.6 | 99.1 | 101.8 | 105.1 | 108.6 | 111.7 | 114.8 |
| 7.0 | 98.4 | 101.0 | 103.8 | 107.3 | 110.9 | 114.1 | 117.2 |
| 7.5 | 100.2 | 102.9 | 105.8 | 109.4 | 113.2 | 116.4 | 119.6 |
| 8.0 | 102.1 | 104.8 | 107.8 | 111.5 | 115.4 | 118.8 | 122.1 |
| 8.5 | 104.0 | 106.7 | 109.7 | 113.6 | 117.6 | 121.1 | 124.6 |
| 9.0 | 106.0 | 108.6 | 111.7 | 115.6 | 119.8 | 123.5 | 127.0 |
| 9.5 | 108.0 | 110.6 | 113.6 | 117.6 | 121.9 | 125.8 | 129.6 |
| 10.0 | 110.0 | 112.6 | 115.6 | 119.7 | 124.1 | 128.2 | 132.2 |
| 10.5 | 112.0 | 114.6 | 117.6 | 121.7 | 126.3 | 130.4 | 134.7 |
| 11.0 | 113.9 | 116.5 | 119.5 | 123.6 | 128.3 | 132.6 | 137.0 |
| 11.5 | 115.8 | 118.4 | 121.4 | 125.6 | 130.4 | 134.7 | 139.1 |
| 12.0 | 117.7 | 120.3 | 123.4 | 127.7 | 132.5 | 136.8 | 141.2 |
| 12.5 | 119.5 | 122.2 | 125.5 | 129.8 | 134.6 | 138.9 | 143.2 |
| 13.0 | 121.3 | 124.1 | 127.4 | 131.8 | 136.6 | 140.9 | 145.0 |
| 13.5 | 122.9 | 125.9 | 129.3 | 133.8 | 138.6 | 142.8 | 146.7 |
| 14.0 | 124.4 | 127.5 | 131.0 | 135.6 | 140.4 | 144.5 | 148.3 |
| 14.5 | 125.8 | 128.9 | 132.6 | 137.2 | 142.0 | 146.0 | 149.6 |
| 15.0 | 126.8 | 130.1 | 133.8 | 138.5 | 143.3 | 147.2 | 150.7 |
| 15.5 | 127.7 | 131.1 | 134.8 | 139.6 | 144.3 | 148.1 | 151.5 |
| 16.0 | 128.5 | 131.8 | 135.6 | 140.4 | 145.1 | 148.8 | 152.1 |
| 16.5 | 129.1 | 132.4 | 136.2 | 141.0 | 145.7 | 149.4 | 152.6 |
| 17.0 | 129.6 | 132.9 | 136.7 | 141.4 | 146.1 | 149.8 | 152.9 |
| 17.5 | 130.0 | 133.3 | 137.0 | 141.8 | 146.5 | 150.1 | 153.1 |
| 18.0 | 130.5 | 133.7 | 137.4 | 142.1 | 146.8 | 150.4 | 153.4 |
| 18.5 | 131.1 | 134.2 | 137.8 | 142.6 | 147.2 | 150.8 | 153.7 |
| 19.0 | 131.7 | 134.7 | 138.3 | 143.1 | 147.8 | 151.3 | 154.2 |
| 19.5 | 132.4 | 135.4 | 139.0 | 143.7 | 148.4 | 151.8 | 154.7 |
| 20.0 | 133.1 | 136.1 | 139.6 | 144.3 | 149.0 | 152.5 | 155.3 |

**Supplement 6**

**Weight calculated according to age group and percentile of 259 girls with TS.**

| Weight (cm) by Age (years) | | | | | | | |
| --- | --- | --- | --- | --- | --- | --- | --- |
| Age | P3 | P10 | P25 | P50 | P75 | P90 | P97 |
| 2.0 | 8.9 | 9.39 | 9.93 | 10.6 | 11.3 | 12.0 | 12.8 |
| 2.5 | 9.5 | 10.03 | 10.63 | 11.4 | 12.2 | 13.0 | 13.9 |
| 3.0 | 10.1 | 10.66 | 11.31 | 12.1 | 13.0 | 13.9 | 14.9 |
| 3.5 | 10.6 | 11.19 | 11.90 | 12.8 | 13.8 | 14.8 | 15.9 |
| 4.0 | 11.0 | 11.66 | 12.42 | 13.4 | 14.4 | 15.6 | 16.8 |
| 4.5 | 11.4 | 12.16 | 12.97 | 14.0 | 15.2 | 16.4 | 17.8 |
| 5.0 | 11.9 | 12.71 | 13.58 | 14.7 | 16.0 | 17.3 | 18.9 |
| 5.5 | 12.5 | 13.28 | 14.22 | 15.4 | 16.9 | 18.4 | 20.1 |
| 6.0 | 13.0 | 13.87 | 14.90 | 16.2 | 17.8 | 19.5 | 21.5 |
| 6.5 | 13.5 | 14.50 | 15.62 | 17.1 | 18.8 | 20.8 | 23.1 |
| 7.0 | 14.1 | 15.16 | 16.39 | 18.0 | 20.0 | 22.2 | 24.8 |
| 7.5 | 14.7 | 15.82 | 17.19 | 19.0 | 21.2 | 23.7 | 26.8 |
| 8.0 | 15.2 | 16.48 | 17.99 | 20.0 | 22.5 | 25.3 | 28.8 |
| 8.5 | 15.7 | 17.14 | 18.81 | 21.0 | 23.8 | 26.9 | 30.9 |
| 9.0 | 16.3 | 17.82 | 19.65 | 22.1 | 25.1 | 28.6 | 32.8 |
| 9.5 | 16.9 | 18.56 | 20.57 | 23.2 | 26.5 | 30.2 | 34.7 |
| 10.0 | 17.5 | 19.39 | 21.59 | 24.5 | 28.0 | 31.9 | 36.6 |
| 10.5 | 18.3 | 20.34 | 22.75 | 25.9 | 29.7 | 33.7 | 38.4 |
| 11.0 | 19.1 | 21.37 | 24.00 | 27.4 | 31.4 | 35.6 | 40.4 |
| 11.5 | 20.0 | 22.46 | 25.33 | 29.0 | 33.2 | 37.5 | 42.4 |
| 12.0 | 20.9 | 23.62 | 26.72 | 30.6 | 35.0 | 39.5 | 44.4 |
| 12.5 | 21.9 | 24.82 | 28.16 | 32.3 | 36.9 | 41.5 | 46.6 |
| 13.0 | 22.9 | 26.05 | 29.59 | 33.9 | 38.8 | 43.5 | 48.7 |
| 13.5 | 23.9 | 27.25 | 30.98 | 35.5 | 40.5 | 45.5 | 50.8 |
| 14.0 | 24.9 | 28.40 | 32.27 | 37.0 | 42.2 | 47.3 | 52.7 |
| 14.5 | 25.9 | 29.45 | 33.42 | 38.3 | 43.6 | 48.9 | 54.5 |
| 15.0 | 26.7 | 30.36 | 34.41 | 39.4 | 44.8 | 50.2 | 55.9 |
| 15.5 | 27.5 | 31.16 | 35.26 | 40.3 | 45.8 | 51.3 | 57.2 |
| 16.0 | 28.2 | 31.88 | 35.99 | 41.1 | 46.7 | 52.2 | 58.2 |
| 16.5 | 28.9 | 32.55 | 36.66 | 41.7 | 47.4 | 53.1 | 59.2 |
| 17.0 | 29.5 | 33.19 | 37.30 | 42.4 | 48.1 | 53.9 | 60.2 |
| 17.5 | 30.2 | 33.83 | 37.94 | 43.1 | 48.9 | 54.7 | 61.2 |
| 18.0 | 30.9 | 34.48 | 38.58 | 43.7 | 49.6 | 55.6 | 62.3 |
| 18.5 | 31.5 | 35.14 | 39.26 | 44.5 | 50.4 | 56.5 | 63.4 |
| 19.0 | 32.2 | 35.82 | 39.95 | 45.2 | 51.2 | 57.5 | 64.6 |
| 19.5 | 32.9 | 36.50 | 40.64 | 45.9 | 52.1 | 58.5 | 65.7 |
| 20.0 | 33.6 | 37.19 | 41.34 | 46.7 | 52.9 | 59.4 | 66.9 |

**Supplement 7**

**BMI calculated according to age group and percentile of 259 girls with TS.**

| BMI (kg/m^2^) by Age (years) | | | | | | | |
| --- | --- | --- | --- | --- | --- | --- | --- |
| Age | P3 | P10 | P25 | P50 | P75 | P90 | P97 |
| 2.0 | 14.3 | 15.0 | 15.7 | 16.4 | 17.1 | 17.8 | 18.6 |
| 2.5 | 14.0 | 14.8 | 15.5 | 16.2 | 16.9 | 17.6 | 18.4 |
| 3.0 | 13.8 | 14.6 | 15.3 | 16.0 | 16.7 | 17.5 | 18.4 |
| 3.5 | 13.6 | 14.4 | 15.2 | 15.9 | 16.6 | 17.4 | 18.3 |
| 4.0 | 13.5 | 14.3 | 15.0 | 15.8 | 16.5 | 17.3 | 18.1 |
| 4.5 | 13.4 | 14.2 | 14.9 | 15.7 | 16.4 | 17.2 | 18.0 |
| 5.0 | 13.4 | 14.2 | 14.9 | 15.6 | 16.3 | 17.1 | 18.0 |
| 5.5 | 13.4 | 14.1 | 14.8 | 15.5 | 16.3 | 17.1 | 18.0 |
| 6.0 | 13.3 | 14.1 | 14.8 | 15.6 | 16.4 | 17.2 | 18.3 |
| 6.5 | 13.3 | 14.1 | 14.9 | 15.7 | 16.5 | 17.5 | 18.7 |
| 7.0 | 13.3 | 14.2 | 15.0 | 15.8 | 16.8 | 17.9 | 19.2 |
| 7.5 | 13.3 | 14.2 | 15.1 | 16.0 | 17.1 | 18.4 | 20.0 |
| 8.0 | 13.3 | 14.2 | 15.2 | 16.2 | 17.5 | 18.9 | 20.7 |
| 8.5 | 13.3 | 14.3 | 15.3 | 16.5 | 17.8 | 19.4 | 21.5 |
| 9.0 | 13.2 | 14.3 | 15.4 | 16.7 | 18.2 | 19.9 | 22.1 |
| 9.5 | 13.2 | 14.3 | 15.6 | 17.0 | 18.6 | 20.4 | 22.7 |
| 10.0 | 13.1 | 14.4 | 15.7 | 17.3 | 19.0 | 20.9 | 23.2 |
| 10.5 | 13.2 | 14.5 | 16.0 | 17.6 | 19.5 | 21.4 | 23.8 |
| 11.0 | 13.2 | 14.7 | 16.2 | 18.0 | 20.0 | 22.0 | 24.4 |
| 11.5 | 13.3 | 14.8 | 16.5 | 18.4 | 20.5 | 22.6 | 25.0 |
| 12.0 | 13.5 | 15.0 | 16.8 | 18.8 | 21.0 | 23.2 | 25.6 |
| 12.5 | 13.7 | 15.3 | 17.0 | 19.2 | 21.5 | 23.7 | 26.1 |
| 13.0 | 13.9 | 15.5 | 17.3 | 19.5 | 21.9 | 24.2 | 26.7 |
| 13.5 | 14.2 | 15.8 | 17.6 | 19.8 | 22.3 | 24.7 | 27.2 |
| 14.0 | 14.5 | 16.1 | 17.8 | 20.1 | 22.7 | 25.1 | 27.6 |
| 14.5 | 14.8 | 16.3 | 18.0 | 20.4 | 23.0 | 25.4 | 27.9 |
| 15.0 | 15.0 | 16.5 | 18.2 | 20.6 | 23.2 | 25.7 | 28.2 |
| 15.5 | 15.2 | 16.6 | 18.4 | 20.8 | 23.4 | 25.9 | 28.3 |
| 16.0 | 15.4 | 16.8 | 18.5 | 20.9 | 23.6 | 26.0 | 28.4 |
| 16.5 | 15.6 | 17.0 | 18.7 | 21.1 | 23.8 | 26.2 | 28.5 |
| 17.0 | 15.8 | 17.1 | 18.8 | 21.3 | 24.0 | 26.4 | 28.7 |
| 17.5 | 16.0 | 17.3 | 19.0 | 21.4 | 24.2 | 26.6 | 28.9 |
| 18.0 | 16.2 | 17.5 | 19.2 | 21.6 | 24.5 | 26.9 | 29.2 |
| 18.5 | 16.4 | 17.7 | 19.3 | 21.9 | 24.8 | 27.2 | 29.4 |
| 19.0 | 16.6 | 17.8 | 19.5 | 22.1 | 25.0 | 27.5 | 29.7 |
| 19.5 | 16.7 | 18.0 | 19.7 | 22.3 | 25.3 | 27.7 | 29.9 |
| 20.0 | 16.9 | 18.1 | 19.8 | 22.5 | 25.5 | 28.0 | 30.1 |
